# Supplementary material for: HIVprotI: an integrated web based platform for prediction and design of HIV proteins inhibitors
Source: J Cheminform. 2018 Mar 9;10:12. doi: 10.1186/s13321-018-0266-y (PMC5845081; doi:10.1186/s13321-018-0266-y)
Supplement: Supplementary file 2 — Additional file 2. Source code of HIVProtI web server. [file 13321_2018_266_MOESM2_ESM.zip › HIVprotI_Source-code/hivprotI/jsmol/jsme/JSME.html]

JSME test page


Your web browser must have JavaScript enabled
in order for this application to display correctly.

# JSME test page

|  |  |  |  |
| --- | --- | --- | --- |
| Turn on antialias | Turn off antialias | Old look | New look |

Turning AA on and off does not work with IE. Try IE 9 or 10 to see the applet with AA on. Unfortunately, AA cannot be turned off in EI9 and IE10.

**public void JME.options(String)**

enables to change applet parameters dynamically from JavaScript. Recognized keywords are :  
**xbutton, noxbutton** - show / hide the X button (even when not visible the X button functionality is accessible through the X and H key shortcuts  
**rbutton, norbutton** - show / hide the R button (to mark connection of substituent with the main scaffold)  
**hydrogens, nohydrogens** - display / hide hydrogens  
**query, noquery** - enable / disable query features  
**autoez, noautoez** - automatic generation of SMILES with E,Z stereochemistry  
**nocanonize** - SMILES canonicalization and detection of aromaticity supressed  
**nostereo** - stereochemistry not considered when creating SMILES  
**reaction, noreaction** - enable / disable reaction input  
**multipart** - possibility to enter multipart structures  
**polarnitro** - prevent automatic conversion of nitro (and similar) groups into nonpolar form  
 **number / autonumber** - possibility to number (mark) atoms  
**depict** - the applet will appear without editing buttons, this is used for structure display only  
**border** (used together with the depict option) - displays a border around depicted molecule
  
**newlook, oldlook** - turn on/off the old Java based JME look (default is off)

**public String getHelpURL()** return the url used to display JSME help page.

**public void alert(String message)** Display an alert box with the look and feel of JSME.

  
  
See also description of the applet's param tag.

|  |  |
| --- | --- |
| Set options: | |
| reaction,noquery,oldlook | Set options |
| Customize the help URL: http://peter-ertl.com/jsme/2013\_03/help.html | Set help URL |

|  |  |  |
| --- | --- | --- |
| Import predefined chemical structure to applet: | | |
| Read JME String | Read Multipart Structure | Read reaction |

|  |  |  |
| --- | --- | --- |
| Export chemical structure from applet: | | |
| Get molfile | Get smiles | Get JME string |
|  | | |

## Known issues

IE9 64 bits in IE8 compatibility mode cannot display lines correctly
